# Predominance of a Drifted Influenza A (H3N2) Clade and Its Association with Age-Specific Influenza Vaccine Effectiveness Variations, Influenza Season 2018–2019

**DOI:** 10.3390/vaccines8010078

**Published:** 2020-02-09

**Authors:** Aharona Glatman-Freedman, Rakefet Pando, Hanna Sefty, Itay Omer, Alina Rosenberg, Yaron Drori, Ital Nemet, Ella Mendelson, Lital Keinan-Boker, Michal Mandelboim

**Affiliations:** 1 Israel Center for Disease Control, Israel Ministry of Health, Tel Hashomer, Ramat Gan 5265601, Israel; 2 School of Public Health, Tel Aviv University Faculty of Medicine, Tel Aviv University, Tel Aviv 6997801, Israel; 3 Central Virology Laboratory, Sheba Medical Center, Israel Ministry of Health, Tel Hashomer, Ramat Gan 5265601, Israel; 4 School of Public Health, University of Haifa, Haifa 3498838, Israel

**Keywords:** influenza vaccine, vaccine effectiveness, influenza A (H3N2), drift

## Abstract

*Background:* Influenza A (H3N2) clade 3C.3a was the predominant influenza virus in Israel throughout the 2018-2019 season, constituting a drift from the influenza A (H3N2) vaccine. We estimated the end-of season vaccine effectiveness (VE) by age, among community patients with influenza-like illness (ILI), considering the hemagglutinin (HA) gene mutations and amino acid substitutions of influenza A (H3N2) viruses detected. *Methods:* Nose-throat samples were analyzed for the presence of influenza virus, type/subtype, and HA gene sequence. HA gene sequences and amino acid substitutions were compared to the influenza A/Singapore/INFIMH-16-0019/2016 (H3N2)-like 2018-2019 vaccine virus, and a phylogenetic tree was generated. Influenza VE against influenza A (H3N2) was estimated using the test-negative design. VE was estimated by age group and by 15 year moving age intervals. *Results:* In total, 90% of the influenza A (H3N2) viruses belonged to the 3C.3a clade, constituting a unique situation in the northern hemisphere. Adjusted all-age influenza A (H3N2) VE was −3.5% (95% CI: −51.2 to 29.1). Although adjusted VEs were very low among infants, children, and young adults, a VE of 45% (95% CI: −19.2 to 74.6) was estimated among adults aged ≥45 years old. *Conclusions:* The higher VE point estimates among older adults may be related to previous exposure to similar influenza viruses.

## 1. Introduction

In recent years, influenza A (H3N2) viruses have presented a significant challenge for vaccine selection. In fact, the recommendation regarding the 2019–2020 northern hemisphere influenza A (H3N2) vaccine component was made one month later than for the influenza A (H1N1)pdm09 and the influenza B vaccine components [1]. This challenge stems, for the most part, from the more frequent genetic changes that the influenza A (H3N2) subtype undergoes, as compared with the influenza A (H1N1) and influenza B viruses [2]. The last few influenza seasons witnessed an influenza A (H3N2) drift, with the emergence of clade 3C.2a during the 2014–2015 season [3], as well as genetic divergence of the 3C.2a A (H3N2) clade into several subclades circulating concurrently, starting from the 2016–2017 season [4]. This rapid process of diversification has raised our concern regarding the ability to select an influenza A (H3N2) vaccine virus that matches the circulating influenza A (H3N2) viruses in all or most countries located in a single hemisphere. The 2018–2019 season in Israel, which was dominated by an influenza A (H3N2) virus for the third time since the 2014–2015 season (Figure S1), provided an opportunity to examine our concern.

Israel has a population of 9.1 million, and is located in the Middle East. Influenza activity in Israel usually lasts from December to March [5], in patterns that are similar to those of temperate northern hemisphere countries [6]. The Israeli Ministry of Health (MOH) endorses yearly influenza vaccination for the entire population, aged 6 months and above [7]. Currently, only the quadrivalent inactivated influenza vaccines (QIV) are available for use in Israel, and are offered free-of-charge through all four Health Maintenance Organizations (HMOs) operating in Israel. A national outpatient influenza surveillance sentinel clinics network was established 22 years ago. It includes primary care clinics run by internists, family physicians, and pediatricians, which are situated in all seven districts of Israel. During the 2018/19 influenza season, 32 sentinel clinics were part of this network. This study estimated the 2018–2019 end-of-season vaccine effectiveness (VE) against laboratory-confirmed influenza A (H3N2) in influenza-like illness (ILI) patients. Age-specific VE analyses were performed, as were virological evaluations.

## 2. Materials and Methods 

### 2.1. Study Period and Population

Combined nose-throat samples were collected from ILI patients visiting the Israel Influenza Surveillance Network (IISN) 32 outpatient sentinel clinics. The study period lasted from September 30th 2018 to March 30^th^ 2019. ILI was defined as a temperature of ≥37.8 °C with at least one of the following symptoms: sore throat, coryza, cough, and muscle ache. Medical teams were granted discretion concerning the inclusion of other symptoms and signs that appeared relevant [3,8]. Demographic and medical information was collected from patients’ medical records, including birth date, gender, symptom onset date, date of nose-throat sampling, chronic medical conditions associated with increased risk for severe influenza and complications, influenza vaccination status for the 2018–2019 season, as well as vaccination date and the type of vaccine administered. For children aged 6 months to 9 years, a second influenza vaccine dose (for those receiving the influenza vaccine for the first time) and its administration date were documented, when applicable.

### 2.2. Laboratory Methods

Samples obtained from ILI patients were tested for influenza virus types and subtypes at the Central Virology Laboratory of the Israel Ministry of Health [4,9]. The viral genomic RNA was extracted using MagNApure 96 (Roche, Mannheim, Germany). The presence of influenza viruses A and B and influenza subtype were examined by real-time reverse transcription polymerase chain reaction (rRT-PCR), using Ambion Ag-Path master mix and TaqMan Chemistry (qRT-PCR), performed in the ABI 7500 Real-Time PCR System (Thermo Fisher Scientific, USA), as previously described [10]. Hemagglutinin (HA) gene sequencing was performed on a subset of viruses selected from different months throughout the 2018–2019 influenza season. The influenza A (H3N2) HA gene was partially amplified using specific primers [3,4,8,10]. The amplified HA RT-PCR products were sequenced as previously described [4]. Genetic analysis was based upon mutations leading to amino acid (AA) substitutions. HA sequences were compared to the sequence of the cell-grown influenza A/Singapore/INFIMH-16-0019/2016 (H3N2)-like virus (the 2018–2019 influenza vaccine reference strain), as well as to the egg-propagated strain, using the Sequencher 5.4 program (Gencodes Corporation, Ann Arbor, MI). A phylogenetic tree was constructed and edited using the FigTree program, version 1.4.2. HA sequences of references influenza viruses used in phylogenetic analysis were obtained from the Global Initiative on Sharing All Influenza Data (GISAID) (https://www.gisaid.org). (Table S1). The 2018-2019 HA sequences used in the phylogenetic analysis were submitted to GISAID under the following accession numbers: EPI337955, EPI337956, EPI337957, EPI337958, EPI337959, EPI337960, EPI337961, EPI337962, EPI337963, EPI337964, EPI337965, EPI337966, EPI337967, EPI337968, EPI337969, EPI337970, EPI341970, EPI337971, EPI337972, EPI337973, EPI337974, EPI337975, EPI337976, EPI337977, EPI337978, EPI337979, EPI337980, EPI337981, EPI341968, EPI341969, EPI341970, EPI347157, EPI337978, EPI365459, EPI365460.

### 2.3. Vaccine Effectiveness Study Design

VE was estimated for individuals ≥6 months of age by means of the test-negative design using the formula (1-OR) x 100 [8]. Sample suitability for VE analysis was determined as previously described [8]. Specifically, samples collected from patients >7 days after the onset of disease and <14 days following influenza vaccination were excluded from analysis. Patients with missing critical data, or unknown vaccination status were excluded as well. A patient was determined vaccinated if the 2018–2019 influenza vaccine was administered ≥14 days before the onset of ILI. For children 6 months to 9 years of age who received the influenza vaccine for the first time in their life, only those receiving two doses, with the second dose being administered ≥14 days prior to the onset of disease, were deemed vaccinated. Those children who were eligible for two vaccine doses, but received only one dose, or the second dose was administered <14 days prior to onset of illness, were considered partially vaccinated, and were excluded from the analysis. 

Influenza A (H3N2)-positive ILI patients were labeled “cases”, whereas influenza-negative ILI patients were labeled “controls”.

VE was calculated for all ages and for specific age groups. In order to gain insight into the VE trend at various ages, adjusted VE was also calculated using moving 15 year intervals, beginning at the first year of life and progressing by 1 year increments [8]. A cubic spline function with knots located at five discrete 15 year age intervals was then applied [8]. 

### 2.4. Statistical Analysis

The Pearson’s *χ*^2^ test was applied for comparison of demographic and medical characteristics between cases and controls. 

A univariate logistic regression model was applied to calculate the odds ratio for crude VE estimates, including the 95% confidence intervals. A multivariate logistic regression model was applied to adjust for age, gender, number of days from disease onset to sampling, calendar month of sample collection, and underlying chronic conditions placing patients at risk for severe influenza or complications. Wherever the number of patients was too small in a particular calendar month, instead of adjusting according to calendar month, adjustment was performed for the high influenza activity period (December, January, February) and low influenza activity period (October, November, March). 

Statistical analyses were performed using SAS Enterprise Guide 7.1 (SAS Institute Inc.) and R version 3.5.2 (R foundation, Vienna, Austria) software.

### 2.5. Ethical Considerations

Influenza surveillance in Israel, including the laboratory confirmation, is implemented under the Israel Public Health Ordinance, and does not entail informed consent.

## 3. Results

### 3.1. Influenza Virus Circulation and Characterization

**Figure 1:**
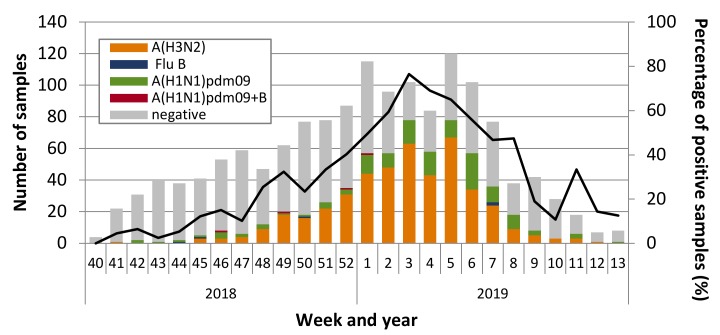
Distribution of influenza types/subtypes by week, among influenza-like illness (ILI) patients presenting to sentinel clinics of the Israel Influenza Surveillance Network (IISN), Israel, September 30, 2018 to March 30, 2019.

During the 2018–2019 study period, 1476 combined nose-throat samples were collected from ILI patients. A total of 594 (40%) were positive for influenza. Of these, 451 (75.9%) were positive for influenza A (H3N2), 134 (22.6%) for influenza A (H1N1)pdm09, 5 (0.8%) for influenza B, and 4 (0.7%) were positive for both influenza A (H1N1)pdm09 and influenza B (Figure 1).

HA gene sequencing analysis was performed on the original samples of 60 influenza A (H3N2)-positive ILI patients presenting to sentinel clinics at various times throughout the 2018-2019 influenza season. Of these, a total of 54 (90%) viruses belonged to the 3C.3a clade, which differed from the 3C.2a1 subclade of the influenza A/Singapore/INFIMH-16-0019/2016 (H3N2)-like virus included in the 2018-2019 influenza vaccine. Only five sequenced influenza A (H3N2) viruses belonged to subclade 3C.2a1, and one belonged to clade 3C.2a.

**Figure 2:**
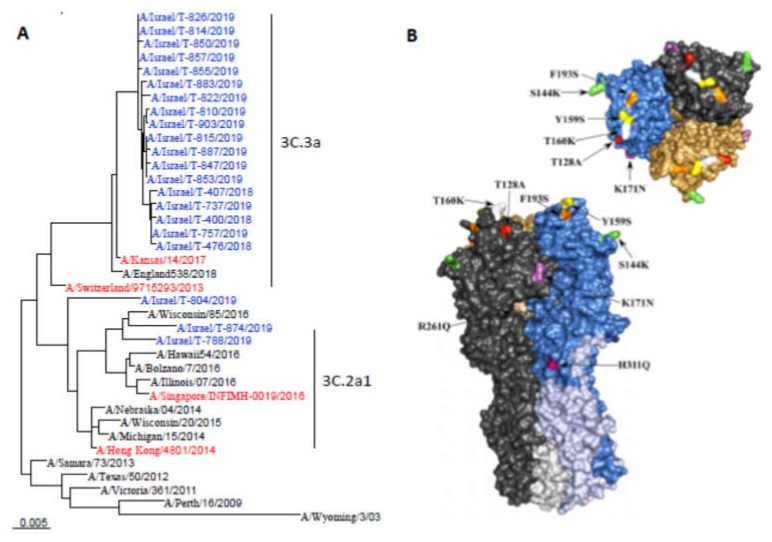
(**A**) Phylogenetic tree of hemagglutinin gene sequences of influenza A (H3N2) viruses obtained from ILI patients, Israel, 2018–2019 season. Relevant influenza vaccine viruses appear in red letters. (**B**) Structure model of a representative influenza A (H3N2) virus belonging to the 3C.3a clade that circulated in Israel during the 2018–2019 season.

Analysis of amino acid (AA) substitutions of the 3C.3a clade influenza A (H3N2) viruses as compared to the cell-grown influenza A/Singapore/INFIMH-16-0019/2016 (H3N2)-like virus, in a representative subset of viruses, is shown in Table S2. A total of 16 substitutions were identified in all viruses. A total of 9 of those 16 substitutions belonged to known antigenic sites that have been associated with antigenic drift mutations [11,12,13,14], that is, antigenic sites A (one substitution), B (four substitutions), C (one substitution), D (one substitution), and E (two substitutions) (Table S2). Additional common AA substitution occurred in several viruses (Table S2). When compared to the egg-propagated vaccine virus, patient samples contained two additional AA substitutions, neither in an antigenic site. Figure 2A presents the phylogenetic tree of representative influenza A (H3N2) viruses. Figure 2B presents a structure model of a representative 2018-2019 influenza A (H3N2) virus belonging to the 3C.3a clade with the identified AA substitutions.

### 3.2. Vaccine Effectiveness against Influenza A (H3N2)

**Figure 3:**
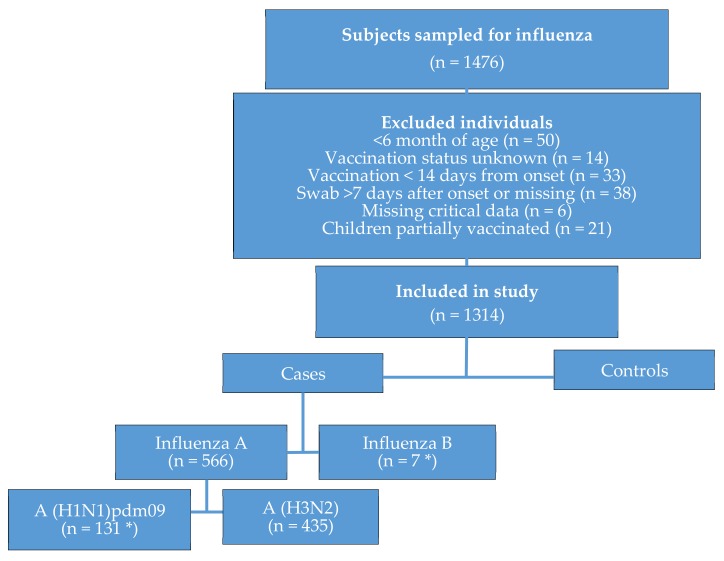
Flowchart of individuals with influenza-like illness presenting at the 2018-2019 sentinel primary care clinics in Israel, with the exclusion of individuals not eligible to be included in the vaccine effectiveness analysis. Asterix symbol (*) denotes the inclusion of four samples coinfected with influenza A (H1N1)pdm09 and influenza B.

A total of 50 individuals were not eligible for VE analysis due to their age (<6 months old), 14 due to unknown vaccination status, 33 due to vaccination <14 days before symptom onset, 38 due to sampling >7 days after symptom onset, 6 due to missing critical data, and 21 children due to partial vaccination (Figure 3). After exclusion of these 162 individuals, 1314 were found eligible for VE analysis (Figure 3); among them, 570 (43%) were positive for influenza. Of these, 435 (76%) were positive for influenza A (H3N2), 131 (23%) for influenza A (H1N1)pdm09, and 7 for influenza B (1%). Three (0.5%) samples were positive for both influenza A (H1N1)pdm09 and influenza B (Figure 3).

As a result of its predominance, VE was estimated against influenza A (H3N2). No VE analysis was carried out for influenza A (H1N1)pdm09 or influenza B due to the small number of cases.

Table 1 presents the characteristics of influenza A (H3N2)-positive ILI patients (cases) and influenza-negative ILI patients (controls). All vaccinated individuals received the 2018-2019 inactivated quadrivalent influenza vaccine (QIV) [15].

Table 2 presents estimated influenza A (H3N2) VE for all ages and for specific age groups. Adjusted VE estimate for all ages was −3.5% (95% CI: −51.2 to 29.1). Adjusted influenza A (H3N2) VE estimates for specific age groups demonstrated that the vaccine was not effective among children and adults <45 years of age. However, for adults aged ≥45 years, the adjusted VE point estimates were in the moderate range. Specifically, the adjusted VE for the 45–64 year old age group was 45.4% (95% CI: −66.8 to 82.1), and for the ≥65 age group it was 46.5% (95% CI: -68.5 to 83.0). Combined adjusted VE estimates for adults aged ≥45 years was 45% (95% CI: -19.2 to 74.6). None of the age-group VE estimates were statistically significant.

In order to gain insight into VE trends at various ages, adjusted VE estimates were calculated according to moving 15 year age intervals, beginning at the first year of life and progressing by 1 year increments [8]. We then applied a cubic spline function with knots located at five discrete 15 year age intervals [8].

**Figure 4:**
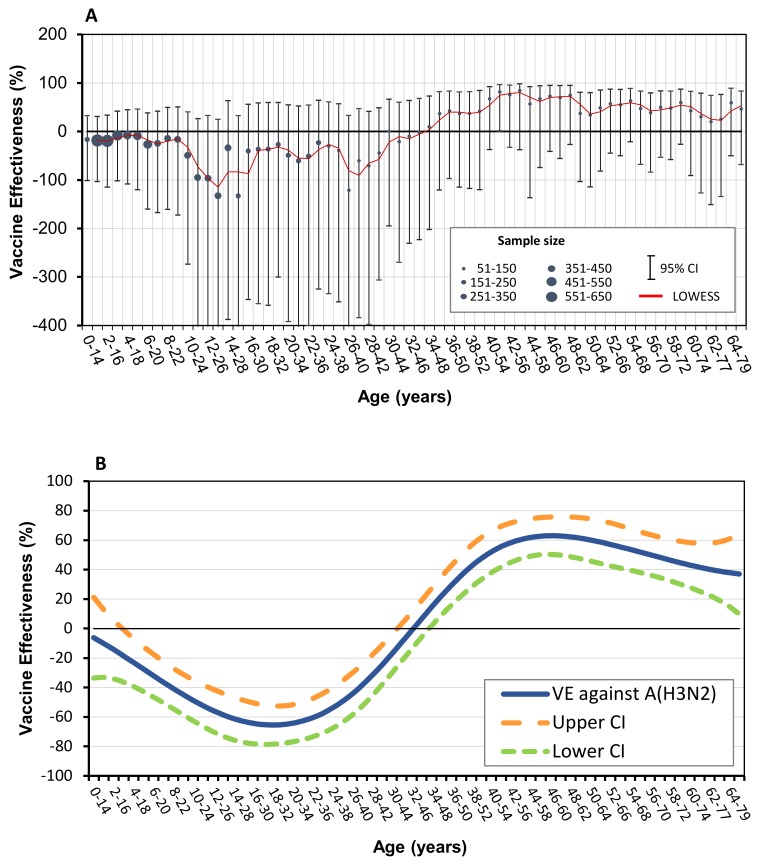
(**A**) Adjusted influenza A (H3N2) vaccine effectiveness (VE) estimates, 2018-2019 season, by age, using moving age intervals of 15 years. Circles represent VE estimates and error bars represent 95% confidence interval (CI) of VE estimates. Solid line (red) represents locally weighted scatterplot smoothing (LOWESS) of dynamic VE point estimates. Circle sizes represent sample sizes. (**B**) Cubic spline applied to adjusted influenza A (H3N2) vaccine effectiveness (VE), 2018-2019 season, using moving age intervals of 15 years. 95% confidence intervals of point estimates are shown.

The adjusted moving influenza A (H3N2) VE estimates are shown in Figure 4A. The age intervals covering ages 0.5 to 47 years demonstrated adjusted VE with negative point estimates that were not statistically significant. The age intervals, starting at the 34–48 years interval and above, showed adjusted VE with positive point estimates. Specifically, the age intervals with the highest adjusted VE were 41–55 and 43–57 years, reaching VE point estimates of 81.5% and 84.3%, respectively. The age intervals of 42–56, 46–60, and 48–62 years old had adjusted VE point estimates between 73.4% and 75.7%. The adjusted VE of the age interval of 41–55 was statistically significant (81.5% (95% CI: 0.9% to 96.6%)), whereas other positive adjusted VE estimates were not statistically significant. The adjusted VE estimates in individuals aged 34 years and over were consistently positive, despite smaller sample sizes as compared with sample sizes of infants, children, and younger adults (Figure 4A). Figure 4B shows the results of applying the cubic spline function to the VE point estimates data presented in Figure 4A.

**Figure 5:**
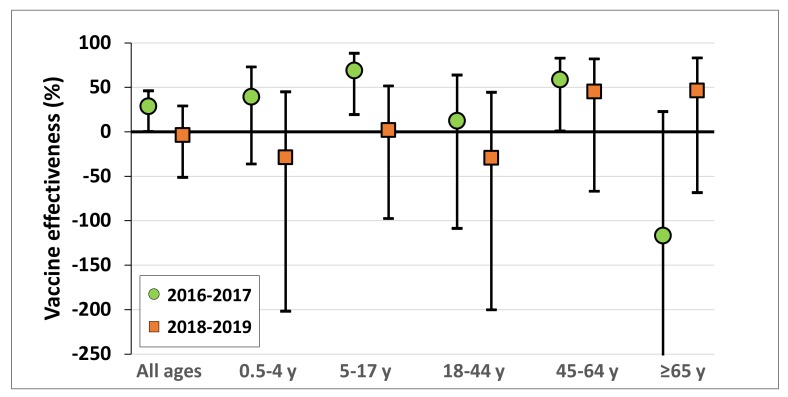
Adjusted influenza A (H3N2) VE of the 2018-2019 and the 2016–2017 influenza seasons for all ages and by age group.

Figure 5 presents the adjusted influenza A (H3N2) VE of the 2018–2019 and 2016–2017 seasons in Israel, both of which were dominated by influenza A (H3N2) [8]. In both seasons, the adjusted VE point estimates were positive in adults of the 45–64 age group [8]. However, although during the 2016-2017 season the adjusted VE had positive point estimates among infants, children, and adolescents [8], they were negative or extremely low during the 2018–2019 season. The adjusted VE for the ≥65 year old age group also differed between the two seasons. Specifically, it had a negative point estimate during the 2016-2017 season [8], whereas it was positive during the 2018–2019 season.

## 4. Discussion

The influenza A (H3N2) was the dominant influenza virus circulating in Israel throughout the 2018–2019 season. Most of these viruses belonged to the 3C.3a clade, a clade different from that of the 2018–2019 influenza A (H3N2) vaccine virus component. The 3C.3a influenza A (H3N2) viruses also differed from influenza A (H3N2) viruses that circulated in most locations in the northern hemisphere, especially in the early and middle parts of the season [16], and had poor antigenic reaction with antibodies elicited by the 2018–2019 influenza A (H3N2) vaccine virus [1].

Prior to the 2018–2019 season, influenza A (H3N2) viruses belonging to the 3C.3 clade circulated in Israel only during the 2013–2014 season [10]. Although the influenza A (H3N2) viruses circulating in Israel during 2018–2019 demonstrated genetic homogeneity within the 3C.3a clade, the 2018–2019 influenza A (H3N2) viruses circulating in European countries and the United States demonstrated genetic diversity within the 2C.2a clade [16,17,18,19,20]. In addition, although the 3C.3a clade constituted the minority of influenza A (H3N2) viruses in the beginning of the season in the USA, its proportion gradually increased throughout the 2018–2019 season [17], reaching 85% of influenza A (H3N2) viruses by the later part of the influenza season [21]. Similarly, the proportion of 3C.3a influenza A (H3N2) increased throughout the season in several Western European countries [1]. This phenomenon indicates that the circulation of influenza A (H3N2) clades can be dynamic even within the same season, and that the circulation of clade 3C.3a in Israel during the 2018-2019 season preceded that of other northern hemisphere countries.

The low all-ages influenza A (H3N2) VE estimate observed in Israel, was consistent with the genetic and antigenic analysis of these viruses. In addition, it was consistent with the all-ages influenza A (H3N2) clade 3C.3a VE estimates in the USA [21], Canada [20], and Europe [22].

Despite the low all-ages influenza A (H3N2) VE estimate in Israel, VE point estimates were higher in adults as compared with younger individuals (Table 2). Specifically, although the influenza A (H3N2) 2018-2019 vaccine was not effective in the 0–4, 5–17, and the 18–44 year old age groups, it demonstrated moderate adjusted VE point estimates in the older age groups (Table 2).

Estimation of VE for predetermined age groups is valuable; however, given the distinct character of such groups, it may not provide enough information regarding the trend of VE at various ages. Using the 15 year moving interval allowed us to evaluate VE at various ages despite the fact that our sample size may be smaller than that of larger countries [8,22,23]. This approach demonstrated the transition from negative to positive influenza A (H3N2) VE point estimates with age (Figure 4A,B).

The difference in VE estimates between the younger and older age groups in our study is interesting. The low VE among infants, children, and young adults can be explained by the vaccine mismatch. However, the higher VE point estimates among older adults may be explained by past exposure to similar influenza A (H3N2) viruses, which may have allowed the mismatched 2018-2019 vaccine to provide them with an immunological boosting effect. Furthermore, it has been proposed that influenza viruses that circulate during a person’s childhood can confer lasting protection against new influenza viruses that belong to a similar phylogenetic group [24]. In this regard, ferrets that were vaccinated with a split-virion influenza vaccine following influenza A (H1N1) A/USSR/90/1977 infection earlier in their life showed less illness after infection with influenza A (H1N1)pdm09 A/California/07/2009 as compared with vaccinated ferrets who were not previously infected with influenza A (H1N1) A/USSR/90/1977 [25]. Moreover, it was recently shown that recurring exposures to influenza A (H3) in humans was associated with higher antibody titers, enhanced antibody affinity, as well as enhanced antibody avidity following influenza vaccination, as compared with individuals not previously exposed [26]. Additional research may be required to further explain the differences in VE point estimates between the younger and older age groups found in the present study.

Although both the 2016-2017 [8] and 2018-2019 seasons in Israel were dominated by the influenza A (H3N2), a drifted influenza A (H3N2) virus circulated only in 2018-2019. The differences and similarities in VE between the 2016–2017 [8] and the 2018-2019 seasons suggest that the influenza A (H3N2) mismatch that occurred in Israel in 2018-2019, affected influenza VE primarily in infants, children, and adolescents. The difference in influenza A (H3N2) VE point estimates for the ≥65 year age group between the two seasons, may be explained by differences in prior exposure to similar viruses/antigens. However, future research may be required to further explain the differences in the adjusted VE results between these two seasons for the ≥65 year age group.

Recent studies by Skowronski et al. [20] and Kissling et al. [22] demonstrated statistically significant negative 2018-2019 influenza A (H3N2) VE estimates among the age ranges of 39–53 years old [20] and 32–54 years old [22], respectively. Skowronski et al. hypothesized that immune imprinting that occurred during childhood towards a common epitope of the hemagglutinin (HA) protected adults that did not receive the 2018–2019 influenza vaccine against the circulating 3C.3a influenza A (H3N2) viruses, whereas the immune response elicited by the 2018–2019 mismatched influenza A (H3N2) vaccine antigen interfered with this protection [20]. However, it is important to recognize that in addition to the imprinting that results from first infection, additional exposures can further shape the immune response towards influenza viruses [27].

In the present study, no statistical significance was noted for any of the age intervals showing negative VE point estimates. The negative VE point estimates observed here among the younger individuals (which were not statistically significant), were similar to smaller declines in VE estimates of younger birth cohorts observed in the study of Skowronski et al. [20]. Thus, the negative VE point estimates observed among the younger individuals in the present study can more likely be ascribed to the mismatch with the 2018-2019 influenza vaccine rather than to prior exposure to influenza viruses.

Children are thought to have the highest rates of illness and complications resulting from influenza [28,29]. In addition, children have been considered to play a key role in the spread of infections [30,31]. Vaccinating children and young adults against influenza was proposed in order to confer indirect protection to the population at large [32]. However, the low VE estimate among infants, children, and adolescents shown in our 2018–2019 analysis may pose a substantial challenge to the prevention of influenza in the community.

This study has several advantages. The predominance of the 3C.3a clade in Israel throughout the 2018–2019 season resulted in a sample size that enabled the analysis of VE trends by age against this clade. Additionally, vaccination status and vaccination dates of ILI patients included in our study were based entirely on medical records’ data. It is also important to realize that Israel is the only country in the Middle East that performs yearly assessments of influenza vaccine effectiveness. Thus, these VE estimates may be relevant also to neighboring countries in the region. In this regard, influenza A (H3N2) circulated during the 2018–2019 season in Lebanon, Jordan, and the Palestinian authority, all of which share borders with Israel [33].

This study was limited by the unavailability of data regarding influenza vaccination in prior seasons. Although studies examining the effect of prior influenza vaccination on current season VE have shown variable results [34,35,36,37,38,39,40,41], most evidence to date suggests that influenza A (H3N2) VE estimates are highest when vaccination is administered during the season that is being evaluated [20,38].

Confidence intervals are used to describe statistical significance of VE estimates. In general, caution should be exercised when VE estimates are not statistically significant, as in such cases, the possibility of ‘no protective effect’ (zero VE) cannot be ruled out [42]. However, the use of statistical significance is being challenged by scientists around the world [43,44]. A discussion regarding this issue is necessary in order to determine how to interpret confidence intervals of VE estimates going forward.

The patterns of genetic evolution and divergence of influenza A (H3N2), as well as their impact on influenza VE, are likely to continue to challenge the selection of a suitable influenza A (H3N2) vaccine component. In the absence of a universal influenza vaccine, we support the concurrent inclusion of influenza A (H3N2) viruses from more than one clade in future influenza vaccines, as was recently suggested by others [45].

## 5. Conclusions

The genetic changes of the influenza A (H3N2) virus will likely continue to present a challenge for the selection of an optimal influenza A (H3N2) vaccine component. New solutions should be considered to overcome this challenge.

## Figures and Tables

**Table 1:** Characteristics of influenza A (H3N2) cases and controls—2018/2019 season.

Variables	Controls (*n* = 744)	Cases (*n* = 435)	Total (*n* = 1179)	*p*-Value ^a^
**Age group (years)**				
0.5-4	252	104	356	>0.01
17-May	136	183	319	
18-44	204	96	300	
45-64	97	29	126	
≥ 65	55	23	78	
**Sex**				
Male	368	218	586	0.83
Female	376	217	593	
**Background Disease**				
No	596	387	983	>0.01
Yes	148	48	196	
**Days between Symptom onset and Swabbing**				
0-1	302	155	457	0.09
04-Feb	379	250	629	
07-May	63	30	93	
**Month of Sample Collection**				
October	107	1	108	>0.01
November	150	18	168	
December	183	110	293	
January	230	135	365	
February	126	69	195	
March	43	7	50	
**Influenza Vaccine**				
No	634	360	994	0.26
Yes	110	75	185	

^a^ Statistical significance for differences between cases and controls.

**Table 2:** Influenza vaccination status and influenza A (H3N2) vaccine effectiveness estimates, 2018-2019 (*n* = 1179).

Age (Years)	Adjustment	Cases			Controls			Adjusted Vaccine Effectiveness	
		%	Vac ^a^	All	%	Vac ^a^	All	95% CI	%
**All ages**	**Crude**	17.2	75	435	14.8	110	744	−65.5 to 12.9	−20.1
**All ages**	**Adjusted ^a^**	17.2	75	435	14.8	110	744	−51.2. to 29.1	−3.5
**0.5-4**	**Adjusted ^b^**	14.4	15	104	8.3	21	252	−201.8 to 5	−28.8
**17-May**	**Adjusted ^b^**	15.3	28	183	12.5	17	136	−97.7 to 51.6	2.1
**18-44**	**Adjusted ^b^**	13.5	13	96	8.3	17	204	−200.2 to 44.5	−29.1
**45-64**	**Adjusted ^b^**	20.7	6	29	24.7	24	97	−66.8 to 82.1	45.4
**65≤**	**Adjusted ^b^**	56.5	13	23	56.4	31	55	−68.5 to 83.0	46.5

^a^ Adjustment for age (as a categorical variable), sex, days from disease onset to swab, and influenza underlying chronic conditions and calendar month of sampling. ^b^ Adjusted for age (as a continues variable), sex, days from disease onset to swab, chronic underlying conditions, and influenza high and low activity periods.

## Supplementary Materials

The following are available online at https://www.mdpi.com/2076-393X/8/1/78/s1. Figure S1: Percentage of influenza-positive samples by type and subtype of all influenza-positive samples obtained from ILI patients presenting to sentinel clinics, by season, Israel 2008-2019 (pandemic influenza season 2009–2010 is excluded). Percentage of influenza A(H3N2) are indicated within the relevant season bars, Table S1: Details of influenza A(H3N2) haemagglutinin sequences used in the phylogenetic analysis, 2018–2019 season, Israel, Table S2: Hemagglutinin (HA) amino acid (AA) substitutions observed in the dominant influenza A(H3N2) 3C.3a clade viruses in Israel, 2018-2019 as compared with the cell-grown influenza A/Singapore/0019/2016-like virus. Yellow cells represent AA substitutions that are common to all viruses; purple cells represent AA substitutions that were found in the majority of viruses; green cells AA substitutions that were found in the minority of viruses. Dots denote AAs that match the influenza A(H3N2) vaccine virus sequence.
