# Supplementary material for: Predominance of a Drifted Influenza A (H3N2) Clade and Its Association with Age-Specific Influenza Vaccine Effectiveness Variations, Influenza Season 2018–2019
Source: Vaccines (Basel). 2020 Feb 9;8(1):78. doi: 10.3390/vaccines8010078 (PMC7157661; doi:10.3390/vaccines8010078)
Supplement: Supplementary file 1 [file vaccines-08-00078-s001.pdf]

vaccines

Supplementary material for the article “Predominance of a drifted influenza A(H3N2) clade and its association with influenza vaccine effectiveness variations by age, influenza season 2018-2019”

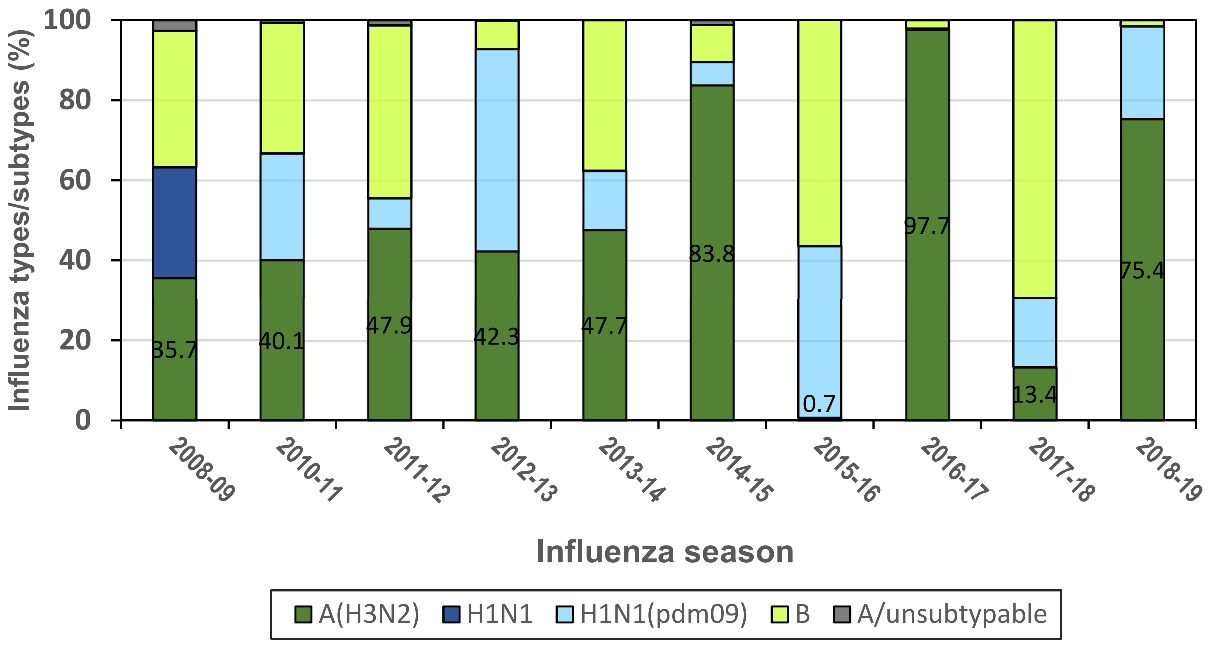

Figure S1. Percentage of influenza-positive samples by type and subtype of all influenza-positive samples obtained from ILI patients presenting to sentinel clinics, by season, Israel 2008-2019 (pandemic influenza season 2009-2010 is excluded). Percentage of influenza A(H3N2) are indicated within the relevant season bars.

Table S1. Details of influenza A(H3N2) haemagglutinin sequences used in the phylogenetic analysis, 2018-2019 season, Israel.

Virus isolate	Sequence source	Accession number	Country	Originating laboratory
A/Singapore/INFIMH-0019/2016	GISAID EpiFlu	EPI239803	Singapore	Ministry of Health,National Public Health Laboratory, Singapore
A/Switzerland/9715293/2013	GISAID EpiFlu	EPI530687	Switzerland	Hopital Cantonal Universitaire de Geneves, Switzerland
A/Kansas/14/2017	GISAID EpiFlu	EPI292575	United States	Kansas Department of Health and Environment, Kansas, United States
A/Wisconsin/85/2016	GISAID EpiFlu	EPI857055	United States	Wisconsin State Laboratory of Hygiene, Virology Unit, Wisconsin, United States
A/Hawaii/54/2016	GISAID EpiFlu	EPI814213	United States	State of Hawaii Department of Health Medical Microbiology Branch, Hawaii, United States
A/Bolzano/7/2016	GISAID EpiFlu	EPI773595	Italy	Istituto Superiore di Sanità, Roma,Rome, Italy
A/Illinois/07/2016	GISAID EpiFlu	EPI752842	United States	Illinois Department of Public Health-Chicago, United States
A/Nebraska/04/2014	GISAID EpiFlu	EPI520356	United States	Nebraska Public Health Lab, Nebraska, United States
A/Wisconsin/20/2015	GISAID EpiFlu	EPI201644	United States	Centers for Disease Control and Prevention, Atlanta, Georgia, United States
A/Michigan/15/2014	GISAID EpiFlu	EPI171757	United States	Centers for Disease Control and Prevention, Atlanta, Georgia, United States
A/Hong Kong/4801/2014	GISAID EpiFlu	EPI539574	China	Government Virus Unit, Hong Kong (SAR)
A/Samara/73/2013	GISAID EpiFlu	EPI460558	Russian Federation	WHO National Influenza Centre, Russian Federation
A/Texas/50/2012	GISAID EpiFlu	EPI377499	United States	Texas Department of State Health Services-Laboratory, Austin, United States
A/Victoria/361/2011	GISAID EpiFlu	EPI349103	Australia	A Melbourne Pathology, Victoria Pde, Australia
A/Perth/16/2009	GISAID EpiFlu	EP1210071	Australia	WHO Collaborating Centre for Reference and Research on Influenza, Melbourne, Australia
A/Wyoming/3/03	GISAID EpiFlu	EPI385944	United States	Not available
A/England/538/2018	GISAID EpiFlu	EPI312041	United Kingdom	Microbiology Services Colindale, Public Health England, London, United Kingdom

Table S2. Hemagglutinin (HA) amino acid (AA) substitutions observed in the dominant influenza A(H3N2) 3C.3a clade viruses in Israel, 2018-2019 as compared with the cell-grown influenza A/Singapore/0019/2016-like virus. Yellow cells represent AA substitutions that are common to all viruses; purple cells represent AA substitutions that were found in the majority of viruses; green cells AA substitutions that were found in the minority of viruses. Dots denote AAs that match the influenza A(H3N2) vaccine virus sequence.

	Clade																								
HA subunit		HA1																			HA2				
AA position		7	9	53	91	121	128	135	138	144	159	160	162	171	193	260	261	290	311	326	77	149	150	155	160
Antigenic site							B			A	B	B		D	B	E	E		C						
A/Singapore/0019/2016	3C.2a1	D	S	D	S	K	T	T	A	S	Y	T	P	K	F	I	R	N	H	K	V	I	E	E	N
A/Israel/T-400/2018	3c.3a	.	.	.	N	N	A	.	S	K	S	K	.	N	S	.	Q	T	Q	R	I	M	G	G	T
A/Israel/R-407/2018	3c.3a	.	.	.	N	N	A	.	S	K	S	K	.	N	S	V	Q	.	Q	R	I	M	G	G	T
A/Israel/T-737/2019	3C.3a	.	.	.	N	N	A	.	S	K	S	K	.	N	S	.	Q	.	Q	R	I	M	G	G	T
A/Israel/T-476/2018	3c.3a	.	.	.	N	N	A	K	S	K	S	K	.	N	S	.	Q	.	Q	R	I	M	G	G	T
A/Israel/T-757/2019	3c.3a	.	.	.	N	N	A	.	S	K	S	K	.	N	S	.	Q	.	Q	R	I	M	G	G	T
A/Israel/T-810/2019	3c.3a	.	.	N	N	N	A	.	S	K	S	K	.	N	S	V	Q	.	Q	R	I	M	G	G	T
A/Israel/T-814/2019	3c.3a	.	.	.	N	N	A	.	S	K	S	K	.	N	S	.	Q	.	Q	R	I	M	G	G	T
A/Israel/T-815/2019	3c.3a	.	.	.	N	N	A	.	S	K	S	K	.	N	S	V	Q	.	Q	R	I	M	G	G	T
A/Israel/T-822/2019	3c.3a	.	.	.	N	N	A	.	S	K	S	K	.	N	S	.	Q	.	Q	R	I	M	G	G	T
A/Israel/T-826/2019	3c.3a	.	.	.	N	N	A	.	S	K	S	K	.	N	S	.	Q	.	Q	R	I	M	G	G	.
A/Israel/T-847/2019	3c.3a	.	.	.	N	N	A	.	S	K	S	K	.	N	S	V	Q	.	Q	R	I	M	G	G	T
A/Israel/T-850/2019	3c.3a	.	.	.	N	N	A	.	S	K	S	K	Q	N	S	.	Q	.	Q	R	I	M	G	G	.
A/Israel/T-853/2019	3c.3a	.	.	.	N	N	A	.	S	K	S	K	.	N	S	V	Q	.	Q	R	I	M	G	G	T
A/Israel/T-855/2019	3c.3a	.	.	.	N	N	A	.	S	K	S	K	.	N	S	.	Q	.	Q	R	I	M	G	G	.
A/Israel/T-857/2019	3c.3a	.	.	.	N	N	A	.	S	K	S	K	.	N	S	.	Q	.	Q	R	I	M	G	G	.
A/Israel/T-883/2019	3c.3a	.	G	.	N	N	A	.	S	K	S	K	.	N	S	.	Q	.	Q	R	I	M	G	G	T
A/Israel/T-887/2019	3c.3a	G	.	.	N	N	A	.	S	K	S	K	.	N	S	V	Q	.	Q	R	I	M	G	G	T
A/Israel/T-903/2019	3c.3a	.	.	N	N	N	A	.	S	K	S	K	.	N	S	.	Q	.	Q	R	I	M	G	G	T
